# A cost-effectiveness analysis of three components of a syndromic surveillance system for the early warning of epidemics in rural China

**DOI:** 10.1186/s12889-015-2475-x

**Published:** 2015-11-14

**Authors:** Yan Ding, Rainer Sauerborn, Biao Xu, Nie Shaofa, Weirong Yan, Vinod K. Diwan, Hengjin Dong

**Affiliations:** Institute of Public Health, Heidelberg University, Heidelberg, Germany; School of Public Health, Fudan University, Shanghai, China; Tongji Medical School, Huazhong University of Science and Technology, Wuhan, China; Institute for Global Health, Karolinska Institutet, Stockholm, Sweden; Center for Health Policy Studies, Zhejiang University School of Medicine, Hangzhou, China

**Keywords:** Cost-effectiveness analysis, Syndromic surveillance system, Epidemic, Rural China

## Abstract

**Background:**

Syndromic surveillance systems (SSSs) collect non-specific syndromes in early stages of disease outbreaks. This makes an SSS a promising tool for the early detection of epidemics. An Integrated Surveillance System in rural China (ISSC project), which added an SSS to the existing Chinese surveillance system for the early warning of epidemics, was implemented from April 2012 to March 2014 in Jiangxi and Hubei Provinces. This study aims to measure the costs and effectiveness of the three components of the SSS in the ISSC project.

**Methods:**

The central measures of the cost-effectiveness analysis of the three components of the syndromic surveillance system were: 1) the costs per reported event, respectively, at the health facilities, the primary schools and the pharmacies; and 2) the operating costs per surveillance unit per year, respectively, at the health facilities, the primary schools and the pharmacies. Effectiveness was expressed by reporting outputs which were numbers of reported events, numbers of raw signals, and numbers of verified signals. The reported events were tracked through an internal data base. Signal verification forms and epidemiological investigation reports were collected from local country centers for disease control and prevention. We adopted project managers’ perspective for the cost analysis. Total costs included set-up costs (system development and training) and operating costs (data collection, quality control and signal verification). We used self-designed questionnaires to collect cost data and received, respectively, 369 and 477 facility and staff questionnaires through a cross-sectional survey with a purposive sampling following the ISSC project. All data were entered into Epidata 3.02 and exported to Stata for descriptive analysis.

**Results:**

The number of daily reported events per unit was the highest at pharmacies, followed by health facilities and finally primary schools. Variances existed within the three groups and also between Jiangxi and Hubei. During a 15-month surveillance period, the number of raw signals for early warning in Jiangxi province (*n* = 36) was nine times of that in Hubei. Health facilities and primary schools had equal numbers of raw signals (*n* = 19), which was 9.5 times of that from pharmacies. Five signals were confirmed as outbreaks, of which two were influenza, two were chicken pox and one was mumps. The cost per reported event was the highest at primary schools, followed by health facilities and then pharmacies. The annual operating cost per surveillance unit was the highest at pharmacies, followed by health facilities and finally primary schools. Both the cost per reported event and the annual operating cost per surveillance unit in Jiangxi in each of the three groups were higher than their counterparts in Hubei.

**Conclusions:**

Health facilities and primary schools are better sources of syndromic surveillance data in the early warning of outbreaks. The annual operating costs of all the three components of the syndromic surveillance system in the ISSC Project were low compared to general government expenditures on health and average individual income in rural China.

## Background

Managing the risks of major outbreaks of communicable diseases and the importation of non-endemic diseases remain important in China, although China is experiencing a rapid epidemiological transition from communicable to non-communicable diseases [1]. Rural China, compared with urban areas, are particularly vulnerable to threats posed by communicable diseases, due to poor hygiene, inadequate sanitation in public places including village clinics and schools, insufficient access to safe drinking water and close human-animal contacts [2–4]. The nationwide infectious disease surveillance system in China is based on confirmed cases [5], whereas the capacities of health facilities in rural China to diagnose and notify communicable diseases are limited [6]. Therefore, a sensitive and convenient early warning surveillance system for infectious disease is urgently needed in rural China.

Syndromic surveillance systems collect non-specific syndromes in the early stages of disease outbreaks. This makes a syndromic surveillance system a promising tool for the early detection of outbreaks. An Integrated Surveillance System in rural China (ISSC project), which was composed of a syndromic surveillance system and the China Information System for Disease Control and Prevention, was implemented from April 2012 to March 2014 in Jiangxi and Hubei Provinces, with the aim of providing an early warning for outbreaks.

A public health surveillance system should be evaluated to determine how well its stated purposes and objectives are met [7]. Existing evaluation studies of public health surveillance systems typically judge quality against a series of attributes (e.g.: timeliness, simplicity, flexibility, acceptability) [8–15]. There is little literature on the costs or effectiveness or cost-effectiveness analysis of infectious disease surveillance and response systems internationally. We found three on cost analysis [16–18] before August 2015, with two narrowed down to syndromic surveillance systems respectively in China [17] and the United States [18]. There was only one on cost-effectiveness analysis, whereas it neither was specifically on a syndromic surveillance system nor in China [19]. In a previous study, we analyzed the costs of data collection at village clinics for the syndromic surveillance system in the ISSC project [17]. We add to the literature by presenting a cost effectiveness analysis of three components of the syndromic surveillance system in the ISSC project, which took, respectively, health facilities (including county hospitals, township hospitals and village clinics), primary schools (including country, township and village levels) and pharmacies (including county and township level) as surveillance units.

### ISSC interventions

The syndromic surveillance system in the ISSC project was an investigatory approach to detect outbreaks of diseases earlier and more comprehensively than might otherwise be possible with the existing infectious disease surveillance system in China. Diseases of concern in this syndromic surveillance system were acute respiratory infectious diseases (e.g., influenza, epidemic cerebrospinal meningitis, measles, chicken pox, mumps), as well as gastrointestinal infectious diseases (e.g., bacillary dysentery, enteritis, viral hepatitis, polio). The syndromic surveillance system in Hubei and Jiangxi was identical, and followed the same steps of operations which included the training of data collectors, data collection, data quality control, data processing and analysis, raw signal detection and signal verification. Data collectors were staff members of the surveillance units and they collected and reported data daily to an internal online database of the ISSC Project. Staff from the County CDC, as well as researchers in this project, assisted by automated data acquisition and generation of statistical signals, were in charge of data quality control, raw signal detection, and signal verification. Jiangxi and Hubei provinces shared a standard operation procedure of the syndromic surveillance system and training materials. Researchers from one university joined the above mentioned activities in Jiangxi, and those from another university joined in Hubei. The concrete operations of the syndromic surveillance system in Jiangxi and Hubei, especially the organizational approaches were different due to the differences of working approaches of the two universities and local staff. Surveillance data included 10-symptom data from health facilities (fever, cough, sore throat, nausea/vomiting, diarrhea, rash, muco-cutaneous hemorrhage, headache, convulsion and disturbance of consciousness), absenteeism data from primary schools (dates, numbers, ages, gender, classes, addresses, reasons for absence, and diseases or syptoms), and sales of selected medicines from pharmacies. All data collectors in Jiangxi Province were trained collectively by researchers from one university, and those in Hubei were trained by researchers from another universities More information on the ISSC project can be found in other studies [20, 21].

### Study sites and setting

This study was performed in Hubei and Jiangxi Provinces, China, in the four study sites of the ISSC project. Table 1 presents the basic characteristics of the two provinces. The ISSC project committee chose the two provinces because 1) they were both more densely populated than low-income regions in western China; 2) both provinces were middle-and low-income regions in China; and 3) the local health authorities showed the willingness to take part in this project.

**Table 1:** Basic characteristics of Hubei and Jiangxi Provinces

Variables		Hubei	Jiangxi	China
Population in 2013 (millions)		58.00	45.00	1,361.00
Population density in 2013 (persons/sq.km)		326.00	262.00	137.00
Proportion of people in rural areas in 2013 (%)		45.49	51.13	46.27
Per capita GDP in 2013 (RMB)		42,613.00	31,771.00	41,908.00
Life expectancy in 2010 (years)^a^		74.90	74.30	74.80
Notifiable infectious diseases in 2012^a^(/10^5^)	Incidence	265.00	206.00	239.00
	Mortality	0.77	0.59	1.24

Footnotes: ^a^Data is quoted from the China Health Statistical Yearbook 2013 [31]; If not specified, data is from the China Statistical Yearbook-2014 [32], published by the National Bureau of Statistics of China.

The sites were termed County A and County B in Hubei Province and County C and County D in Jiangxi Province. Each province had one county with higher annual income per capita of rural residents in 2011 (County B in Hubei: 8,049 RMB and County C in Jiangxi: 7,926 RMB) and smaller incidence of infectious diseases in 2011(County B in Hubei: 270/10^5^ and County C in Jiangxi: 307/10^5^) compared with its average level, and the other county with the opposite characteristics (County A in Hubei: 7,684 RMB, 625/10^5^ and County D in Jiangxi: 7,400 RMB, 668/10^5^).

Table 2 shows the numbers of surveillance units of the syndromic surveillance system in the ISSC project. County C and County D in Jiangxi Province had more surveillance units at township level than those in Hubei Province. The reasons are: 1) in general one town in County C or County D covered less population than that in County A or County B; 2) the ISSC project covered a similar size of population in each county (around 150,000 people), therefore covered more towns in County C and D; and 3) in general each town had one township hospital, one primary school and one to two pharmacies.

**Table 2:** Surveillance units for the formal implementation of the ISSC

	Level	Hubei			Jiangxi			Total
		A	B	Subtotal	C	D	Subtotal	
Health facilities	County	1	1	2	1	1	2	4
	Township	3	3	6	6	9	15	21
	Villages	76	70	146	69	74	143	289
Pharmacies	County	7	4	11	5	4	9	20
	Township	4	8	12	14	7	21	33
Primary schools	County	4	2	6	5	4	9	11
	Township	4	3	7	6	10	16	23
	Village	7	13	20	15	22	37	57
Total		106	104	210	121	131	252	462

## Methods

The time horizon of this study was 15 months, covering the formal implementation period of the ISSC from April 1, 2012, to June 30, 2013. The central measures of the cost-effectiveness analysis of the three components of the syndromic surveillance system were: 1) the costs per reported event, respectively, at the health facilities, the primary schools and the pharmacies; and 2) the operating costs per surveillance unit per year, respectively, at the health facilities, the primary schools and the pharmacies.

### Sampling strategy

The sampling strategy of the ISSC project for the surveillance units were that at county level, one county level hospital and all registered county level primary schools were selected, and the number and locations of pharmacies was decided by the amount and distribution of pharmacies in each county, and that at township level, the ISSC adopted stratified cluster sampling with towns as basic sample units. In addition, sampled towns were adjacent, which was a requirement of the models in spatial cluster analysis. Therefore, it was not a randomized sampling. Willingness and capacity in performing the surveillance were also taken into consideration when finalized surveillance units for the ISSC project. This study adopted a non-randomized cluster sampling strategy following the ISSC project.

### Cost data

The cost analysis part was performed from the project managers’ point of view. All cost data were reported in Chinese currency (Renminbi; RMB), and costs were not discounted or adjusted for inflation due to the short time horizon.

Total costs included set-up costs (system development and training) and operating costs (data collection, quality control and signal verification). Cost data included staff time, the annual depreciation and opportunity costs of computers and overhead allocation. The staff time costs were measured by multiplying the number of full time employment equivalents with annual salaries and benefits, as suggested in several previous studies [16, 22–24]. The costs of the depreciation and opportunity costs of the computers for the surveillance were measured by annualizing the computer purchasing costs according to their usage life and depreciation rate. Overhead costs were allocated to the surveillance with the percent of staff time as an allocation basis. More details on the cost analysis of the syndromic surveillance system were available in a previous study [17].

Primary data were collected during a three-month field trip from May to July 2013 with self designed facility and staff questionnaires, respectively, for the health facilities, the primary schools, the pharmacies, the county centres for disease control and prevention (County CDC) and the two universities who supported the implementation of the ISSC project. Facility questionnaires collected information on the size of population served, staff, computers, office space, income and expenditures, and the way in which the syndromic surveillance system had been implemented. The staff questionnaire for those from all the facilities was identical. It collected individual information on the daily working time, and the time spent on the surveillance both before and after the staff got familiar with the tasks involved in the surveillance. In total, we collected 369 valid facility and 477 staff questionnaires. Also, we collected daily timesheets of researchers from the two Chinese universities who facilitated the implementation of the surveillance in order to quantify time from the researchers’ side.

Data from the survey of our questionnaires were first entered into Epidata 3.02 (the EpiData Association, Odense, Denmark), and then exported to Stata (StataCorp LP, Texas, USA) for descriptive analysis.

### Effectiveness data

The effectiveness was expressed by indicators of the outputs of the syndromic surveillance system, which were the number of reported events, raw signals and verified signals at, respectively, the health facilities, the primary schools and the pharmacies. Table 3 presents the definitions of those outputs. The number of reported events were basic data of the surveillance which were grouped into syndrome data. The syndromic surveillance system then based on those syndrome data detected raw signals for the early warning of infectious disease outbreaks. Verification of raw signals, either by checking data, telephone investigation, field investigation or laboratory tests, were followed to identify real outbreaks from background noises.

**Table 3:** Definitions of the outputs of the syndromic surveillance system

Outputs	Three types of surveillance units		
	Health facilities	Primary schools	Pharmacies
A reported event	Any report concerning an outpatient with at least one of the ten targeted symptoms (fever, cough, sore throat, nausea/vomiting, diarrhea, rash, muco-cutaneous hemorrhage, headache, convulsion and disturbance of consciousness)	Each absence of students at the primary schools	A reported sale of one unit pack of medicine as defined by the accompanying patent instructions
Syndromes	4 syndromes: acute respiratory infection (patients with fever and either cough or sore throat), influenza-like illness (patients with temperature ≥38 °C and with either cough or sore throat), fever gastro syndrome (patients with fever and with either diarrhea or nausea/vomiting), and fever and rash (patients with both fever and rash)	Clusters of absence by classes	5 syndromes: Compound cold medicines, antitussive, antibiotics, febrifuge and antidiarrheal agents
A raw signal	A data cluster detected by the automated statistical analysis of the syndromic surveillance system or by manual detection of the data management personnel which is a suspect of an infectious disease outbreak.		
A confirmed signal (an outbreak)	A disease cluster with explicit agents and evidence of transmission which might represent or develop to a true outbreak in the absence of early intervention		

The reported events were collected from all the surveillance units by logging in the internal database of the ISSC project. The number of raw signals and their verification information were received from the local CDCs by collecting all forms for signal verification and also epidemiological investigation reports.

Reported events were imported from the ISSC internal database to Stata (StataCorp LP, Texas, USA) for descriptive analysis. Signals were sorted manually according to the content of the forms for signal verification and the epidemiological investigation reports.

## Results

### Reported events to the syndromic surveillance system

**Fig. 1:**
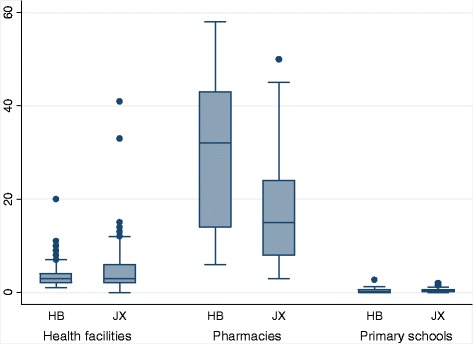
The average numbers of daily reported events per surveillance unit at the three data sources in Hubei (HB) and Jiangxi (JX) provinces. Footnote: Ten outliers from pharmacies were not shown in this graph, range (67, 341); 5 in HB, 5 in JX

Figure 1 presents the distribution of average numbers of daily reported events per surveillance unit, respectively, at the health facilities, the primary schools and the pharmacies in Hubei and Jiangxi Provinces. Huge differences existed within the three groups and also between Hubei and Jiangxi provinces: 1) the pharmacies on average reported the highest events per day per unit (P50 = 39 in Hubei, P50 = 17 in Jiangxi), with their median value dozens of times higher than that of the health facilities (P50 = 3 in both Hubei and Jiangxi), and of the primary schools (P50 = 0 in both Hubei and Jiangxi); 2) the daily number of reported events per unit within the pharmacies ranged widely in both provinces: (P25 = 22, P75 = 63 in Hubei, P25 = 10, P75 = 45 in Jiangxi), followed by those at the health facilities (P25 = 2, P75 = 4 in Hubei, P25 = 2, P75 = 6 in Jiangxi), and then those at the primary schools (P25 = 0, P75 = 1 in both Hubei and Jiangxi); and 3) the outliers of the numbers of daily reported events at the health facility group in Jiangxi province was unusual: among the 6 outliers which were extremely large numbers of daily reported events, four were from the village clinics, two from the two county hospitals but zero from the twelve township level hospitals. In general, the scales of the health facilities, the primary schools and the pharmacies decreased from the county level to township level and final the village level. It would not be surprising if extremely large numbers of daily reported events were mainly from upper levels such as country and township hospitals. It was unexpected that four outliers were from village clinics not township hospitals.

### Raw and verified signals detected based on the reported events

Raw signals detected by the surveillance system were mainly related to fever or influenza. Five signals were confirmed as outbreaks, of which two were chickenpox, two were influenza and the other one was mumps. Table 4 presents the numbers of signals detected by, respectively, health facilities, primary schools and pharmacies. The syndromic surveillance system detected 40 raw signals in total during the 15 months’ operation period. The signals detected had four main characteristics. First, the number of raw signals in Jiangxi Province was 9 times of that in Hubei Province. Second, the only four signals in Hubei Province were all confirmed signals generated by the data reported by a single county primary school, and the 36 raw signals in Jiangxi Province had one confirmed and 23 followed up. Third, flowing-up signals means that more attention were paid to gather further data from the surveillance units which had reported the abnormal data and generated the signals. The personnel of related surveillance units and the County CDC took preventive measures as reactions to the 23 signals followed up in Jiangxi Province. Those activities may have preventive effect but we were not able to quantify it. Fourth, the number of raw signals from the health facilities (which had 314 surveillance units) equaled that from the primary schools (91 surveillance units), and was 9.5 times of that from the pharmacies (53 surveillance units).

**Table 4:** Numbers of signals from the health facilities, schools and pharmacies

Sources	Signals	Hubei	Jiangxi	Total
Health facilities	Excluded as not relevant	0	8	8
	Suspected relevant and followed up	0	10	10
	Confirmed as outbreaks	0	1	1
	Total signals received	0	19	19
Primary schools	Excluded as not relevant	0	2	2
	Suspected relevant and followed up	0	13	13
	Confirmed as outbreaks	4	0	4
	Total signals received	4	15	19
Pharmacies	Excluded as not relevant	0	2	2
	Suspected relevant and followed up	0	0	0
	Confirmed as outbreaks	0	0	0
	Total signals received	0	2	2
All	Excluded as not relevant	0	12	12
	Suspected relevant and followed up	0	23	23
	Confirmed as outbreaks	4	1	5
	Total signals received	4	36	40

### Costs of the syndromic surveillance system

The set-up costs of the syndromic surveillance system in the four counties in Hubei and Jiangxi provinces were 1,459,169 RMB and the annual operating costs of the system in 2012 in the four counties was 1,086,455 RMB (the exchange of Chinese currency was 6.31 RMB for 1 US$ according to the World Bank in 2012 [25]). Table 5 presents the operating costs of the syndromic surveillance system. The costs of personnel time for data collection dominated the costs in Hubei Province, whereas the annual overhead costs were more than half the operating costs in Jiangxi Province.

**Table 5:** Costs of the syndromic surveillance system in RMB

Cost categories	Units	Hubei	Jiangxi	Total
Annual costs of staff for data collection In 2012	Health facilities	113,214	153,477	269,967
	Primary schools	21,642	53,510	75,152
	Pharmacies	13,225	20,069	33,294
	Subtotal (%)	151,357 (52.6)	227,056 (28.4)	378,413 (34.8)
Costs of logs for data collection		11,847 (4.1)	15,239 (1.9)	27,086 (2.5)
Costs of staff for daily management of data collection	By researchers	16,432	45,510	61,942
	By CDC staff	6,186	90,000	96,186
	Subtotal (%)	22,618 (7.9)	135,510 (17.0)	158,128 (14.6)
Costs for fieldworks for the management of data collection	By researchers	9,570	31,446	41,016
	By CDC staff	-	-	-
	Subtotal (%)	9,570(3.3)	31,446 (3.9)	41,016 (3.8)
Costs for signal verification	By researchers	0	8,265	8,265
	By CDC staff	132	0	0
	Subtotal (%)	132 (0.0)	8,265 (1.0)	8,397 (0.8)
Annual depreciation and opportunity costs of computers in 2012	Health facilities	21,474	21,658	43,132
	Primary schools	3,274	3,430	6,704
	Pharmacies	2,501	2,834	5,335
	Local CDCs	3,956	1,340	5,296
	Subtotal (%)	31,205 (11.8)	29,262(3.7)	60,467 (5.6)
Annual costs of computer operation in 2012	Health facilities	1,984	3,324	5,308
	Primary schools	2,385	3,445	5,830
	Pharmacies	3,432	3,267	6,699
	Subtotal (%)	7,801 (2.7)	10,036(1.3)	17,837 (1.6)
Annual overhead costs in 2012	Health facilities	21,196	40,081	61,277
	Local CDCs	32,275	301,559	333,834
	Subtotal (%)	53,471 (18.6)	341,640 (42.8)	395,111 (36.4)
Total		288,001 (100.0)	798,454 (100.0)	1,086,455 (100.0)

Footnotes: in 2012, the exchange of Chinese currency was 6.31 RMB for 1 US$ according to the World Bank [25].

The general government expenditure on health in the two counties in Jiangxi Province was around 220 million RMB according to our investigation. We did not have data on that in the two counties in Hubei Province. Nevertheless, we did a proxy comparison. The annual operating costs of the syndromic surveillance system in the four counties in Hubei and Jiangxi Provinces was around 0.45 percent of the general government expenditures on health in 2012 in the two counties in Jiangxi Province.

### Costs per reported event and annual operating costs per surveillance unit

Table 6 shows the costs per reported event and the costs per surveillance unit in the three surveillance groups in Hubei and Jiangxi Provinces. As a whole, the three types of surveillance units in Jiangxi had both higher costs per reported event and higher costs per surveillance unit compared with their counterparts in Hubei Province. When checked the data by the types of surveillance units, the primary schools had the highest cost per reported event, followed by the health facilities and finally the pharmacies in both Hubei and Jiangxi Provinces. The pharmacies, in contrast to their lowest cost per reported event, had the highest cost per surveillance unit per year, followed by the health facilities, and finally the primary schools, also in both Hubei and Jiangxi Provinces.

**Table 6:** Costs per reported event and costs per surveillance unit

	Costs per reported event (RMB)		Costs per surveillance unit (RMB)	
	Hubei	Jiangxi	Hubei	Jiangxi
Health facilities	1.4	2.0	1,688	4,007
Primary schools	4.3	10.7	1,499	2,866
Pharmacies	0.0	0.1	1,970	5,780

Footnotes: in 2012, the exchange of Chinese currency was 6.31 RMB for 1 US$ according to the World Bank [25]

## Discussion

By tracing inputs to all related activities due to the syndromic surveillance system in the ISSC Project, we identified, measured and quantified the costs of the syndromic surveillance system including the set-up costs and the operating costs. By tracking the outputs of the three components of the surveillance, we described the performance of the the health facilities, the primary schools and the pharmacies in giving early warning for infectious disease outbreaks, and presented the connection between reported events, raw signals and verified signals. By comparing the operating costs and the outputs, we learned the annual operating costs per surveillance unit and the costs per reported event, respectively, at the health facilities, the primary schools and the pharmacies.

Some studies used the number of signals as an indicator for effectiveness of a infectious disease surveillance system [26], whereas more commonly the number of deaths averted or cases of infectious diseases averted due to a surveillance system are used as indicators for effectiveness of an infectious disease surveillance system [19, 27]. The surveillance system had detected influenza, mumps and chicken pox during the study horizon and those were mild diseases which were cured within days without mortality. Therefore, we did not use deaths averted as an effectiveness indicator. This study did not have enough data to model how many cases of influenza, mumps and chicken pox were averted due to the early warning of the surveillance, as the existing national notifiable infectious disease reporting system did not have proper data on cases of influenza, mumps and chicken pox. Influenza and mumps are classified as group C infectious diseases which could be reported to the existing national notifiable infectious disease reporting system but not as a requirement, and chicken pox had not yet been included into the notifiable infectious disease groups when this study was drafted [5].

Our study shown that pharmacies with the highest number of reported events had both the lowest numbers of raw and verified signals. Reported events may not be a sufficiently favorable measure for future studies as an effectiveness indicator for outbreaks detection. They, on the other hand, are the basic data for the syndromic surveillance system. Their quality should have a not neglectable influence on the effectiveness of a syndromic surveillance system. Some study indicated the concern about data quality in this project [28, 29]. This study, by identifing and describing the variances on daily numbers of reported events per surveillance unit within the three types of surveillance units and between the two provinces, could be used to explore likely sources of imperfect data collection.

Our study shows that the health facilities and the primary schools performed better in the early warning of outbreaks during the 15 months’ implementation period judged by the raw signals as well as the confirmed ones. This is identical to a performance evaluation of outbreak detection based on modeling [30]. Considering in addition that the pharmacies had the highest annual surveillance cost per unit, the pharmacies seem do not have advantages in the early warning of outbreaks compared to the primary schools and the health facilities. We, however, do not deny the pharmacies’ supplementary effect in the early warning of outbreaks. One, however, should be cautious in interpreting the effectiveness of pharmacies: 1) the number of pharmacies as surveillance units was far less than that of primary schools as well as that of health facilities; and 2) not all pharmacies in the four counties were surveillance units and the pharmacies as surveillance units may not be representative. It would be good if future study could recruit all pharmacies in at least one county to explore the effectiveness of pharmacies in early warning.

Jiangxi and Hubei Province had huge differences in the pattern and number of reported events as well as numbers of raw and confirmed signals. We do not have evidence that those differences are related to the differences of characteristics of the four counties and the two provinces.

The operating costs of the syndromic surveillance system in, respectively, Hubei and Jiangxi Provinces have huge differences both in terms of the total operating costs and the main components. By tracing activities and inputs we calculated the costs. Therefore, the results were context specific.

The annual income per person in rural China in 2012 was 7,917 RMB [31], and the annual operating cost per unit in the three surveillance units in Hubei and Jiangxi Provinces ranged from 1,499 to 5,780 RMB in 2012. Therefore, the annual operating cost per unit ranged from 19 % to 73 % of the annual income of one person in rural China. The annual operating costs of the syndromic surveillance system in the ISSC Project were low compared to general government expenditures on health and average individual income in rural China.

Our study has a short time horizon during which the surveillance system may not be able to show its full potential in the early warning of outbreaks. Different threshold of triggers can influence the number of raw signals detected which weakens the justification of taking the number of raw signals as an indicator for effectiveness. It is difficult to measure the impact of an infectious disease surveillance and response system [19]. As an exploration through a clear descriptive analysis, this study can provide some reference for managers of syndromic surveillance systems.

## Conclusions

Health facilities and primary schools are better sources of syndromic surveillance data in the early warning of outbreaks. The annual operating costs of all the three components of the syndromic surveillance system in the ISSC Project were low compared to general government expenditures on health and average individual income in rural China.
